# Behind the Robot’s Smiles and Frowns: In Social Context, People Do Not Mirror Android’s Expressions But React to Their Informational Value

**DOI:** 10.3389/fnbot.2018.00014

**Published:** 2018-04-24

**Authors:** Galit Hofree, Paul Ruvolo, Audrey Reinert, Marian S. Bartlett, Piotr Winkielman

**Affiliations:** ^1^Department of Psychology, University of California, San Diego, San Diego, CA, United States; ^2^Department of Engineering, Franklin W. Olin College of Engineering, Needham, MA, United States; ^3^Department of Industrial Engineering, Purdue University, West Lafayette, IN, United States; ^4^Institute for Neural Computation, University of California, San Diego, San Diego, CA, United States; ^5^Department of Psychology, SWPS University of Social Sciences and Humanities, Warsaw, Poland

**Keywords:** facial expressions, emotions, human-robot-interaction, android, affect, electromyography, embodiment

## Abstract

Facial actions are key elements of non-verbal behavior. Perceivers’ reactions to others’ facial expressions often represent a match or mirroring (e.g., they smile to a smile). However, the information conveyed by an expression depends on context. Thus, when shown by an opponent, a smile conveys bad news and evokes frowning. The availability of anthropomorphic agents capable of facial actions raises the question of how people respond to such agents in social context. We explored this issue in a study where participants played a strategic game with or against a facially expressive android. Electromyography (EMG) recorded participants’ reactions over zygomaticus muscle (smiling) and corrugator muscle (frowning). We found that participants’ facial responses to android’s expressions reflect their informational value, rather than a direct match. Overall, participants smiled more, and frowned less, when winning than losing. Critically, participants’ responses to the game outcome were similar regardless of whether it was conveyed via the android’s smile or frown. Furthermore, the outcome had greater impact on people’s facial reactions when it was conveyed through android’s face than a computer screen. These findings demonstrate that facial actions of artificial agents impact human facial responding. They also suggest a sophistication in human-robot communication that highlights the signaling value of facial expressions.

## Introduction

**Figure 1:**
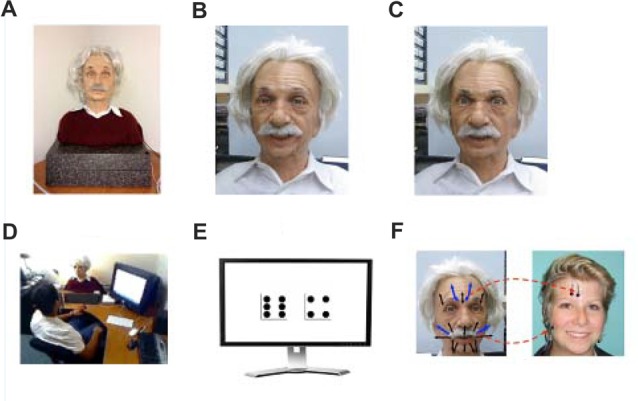
Explanation of the experiment set-up. **(A)** The android as seen by the participant. **(B)** Android smiling. **(C)** Android frowning. **(D)** Arrangement of android and computer. **(E)** Display of a dice roll. **(F)** Location of electromyography (EMG) electrodes and its relation to the android’s face.

How do people respond to anthropomorphic agents with the ability to engage in human-like facial action? This specific question, which we empirically address in the current article, is nested within a growing interest by psychologists, cognitive scientists and neuroscientists in robots and androids as tools to exploring the function of the human mind and brain (Sanchez-Vives and Slater, 2005). For example, studies with such artificial agents have revealed insights into basic mechanisms of perception (Saygin et al., 2012), emotion (Breazeal, 2003), imitation (Hofree et al., 2014, 2015), and attributions of experience and agency (Gray and Wegner, 2012). Recent advances in technology, leading to creation of highly realistic androids, have extended the scientific possibilities to ask new questions that have both theoretical importance as well as practical relevance, given the increasing societal use of such androids (Tanaka et al., 2007; Beasley, 2012; Gratch et al., 2013). One such question is the impact of anthropomorphic appearance of such agents, on human emotional reactions and experience. This question drives research inspired by the “uncanny valley” hypothesis (Mori, 1970), which explores the emotional consequences of android’s increasing similarity to human appearance (Ishiguro, 2006; MacDorman and Ishiguro, 2006; Cheetham et al., 2011; McDonnell et al., 2012; Carr et al., 2017). Importantly, some of the advanced androids have not only anthropomorphic shapes, but can also generate human-like facial displays with realistic movement dynamics. One example is the android (Hanson’s Einstein) shown in Figure 1, more fully described shortly, which we used in the current project. The availability of such agents allows us to ask new and important questions about the impact of expressive signals provided by such realistic agents. This issue has fresh practical relevance as androids with human-like expressive capabilities enter the real the world and become available to the research community. It is also theoretically important to understand the psychological and physiological processes by which expressive tools of a realistic android (e.g., its “face”) drive human responses. One particularly important question here is how the impact of facial displays is modified by a larger social and personal context—a key issue in social robotics, psychology and neuroscience. We elaborate on this shortly, but first it is useful to provide some background on the topic of facial actions and their functioning in social contexts.

### Facial Actions

Facial actions (such as smiling, frowning, or yawning) represent an important part of human social life (Darwin et al., 1872; Ekman and Oster, 1979). Perception as well as production of facial actions can reflect a variety of processes that range from implicit, fast, and effortless, to explicit, deliberative and effortful (Niedenthal and Brauer, 2012). As such, facial actions can carry a variety of information. At the most basic level, facial actions can be simple, non-communicative motor reactions (as when people spontaneously yawn). Of course, the most socially interesting facial actions, typically called “emotional expressions,” inform the observer of the displayer’s underlying emotional states, behavioral dispositions and communicative intentions (Keltner and Haidt, 1999; Horstmann, 2003; Russell et al., 2003). For this reason, the commonly used term “emotional facial expression” is a bit of a misnomer, since it implies the presence of an internal emotion state (along with its external manifestation), rather than a variety of states that facial actions can reveal. These distinctions are important to keep in mind when considering how people may respond to facial actions of realistic androids: the topic of the current article.

### People’s Reactions to Facial Actions, With and Without Social Context

Given the importance and variety of social meanings associated with facial actions, much research is devoted to understanding their function in different contexts. Here, one key distinction is between research that examines people’s reactions to facial actions in contexts that are relatively simple and do not require much interpretation, and research that examines reactions to facial actions embedded in a richer social context.

Research that examines reactions to facial actions in relatively context-poor settings, suggests that perceivers’ responses are driven by two basic factors. One is a motor match, also called “mimicry”, where participants recreate the observed movement in their own face. The other is a valence match, where participants respond to the perceived affect of the face. Though it is possible to separate the role of these factors, in many simple situations, the motor match and valence match lead to the same responses (Moody et al., 2007; Neumann et al., 2014). Thus, when people see smiles, they spontaneously respond with smiles, matching the perceived facial action and positive valence. Similarly, when people see frowns, they spontaneously respond with frowns, matching perceived action and negative valence. These responses can be quick and spontaneous (Dimberg, 1982) and occur with minimal input (Dimberg et al., 2000; Bornemann et al., 2012). Mechanistically, the underlying processes probably reflect the engagement of basic mechanisms underlying mirroring (Carr et al., 2003; Mukamel et al., 2010) and rapid extraction of stimulus value (Murphy and Zajonc, 1993; Winkielman et al., 2005).

Critically, similar mechanisms appear to operate when humans respond to facial actions generated by a realistic android. We demonstrated this in a previous study in which people watched Hanson’s Einstein perform happy and angry facial expressions (Hofree et al., 2014). The procedure was simple—in the first block of the study, participants were instructed to simply watch the android (spontaneous observation); and in the second block, people were instructed to deliberately mimic the android. Participants’ facial responses were measured using electromyography (EMG) over the zygomaticus major (used in smiling) and corrugator supercilii (used in frowning) muscles. We then conducted analyses of EMG amplitude data and synchronization data, comparing the time course of human EMG activity to the voltages supplied to the android’s facial motors. These analyses found robust mirroring in both the spontaneous and deliberate blocks. That is, participants smiled to the android’s smile and frowned to the android’s anger, tightly synchronizing their motor movements with the android’s facial actions. Overall, this study shows that in a default, “mere observation” context, a realistic android can drive basic, spontaneous human facial reactions in a fashion that resembles canonical “mirroring” responses to human faces.

However, as mentioned, reactions to facial actions also depend on social context (Hess and Fischer, 2013; Seibt et al., 2015). For example, people will mimic the counterpart’s emotions in a cooperative setting and show the reverse action in a competitive setting (Lanzetta and Englis, 1989). Furthermore, people show reduced mimicry reactions to individuals that are disliked, belong to out-groups, or have different goals (McIntosh, 2006; Bourgeois and Hess, 2008; Likowski et al., 2008; Leighton et al., 2010). The fundamental impact of contextual cues was recently demonstrated in a study manipulating social power of the perceiver and the displayer of facial actions (Carr et al., 2014). The results showed that perceivers high in social power exhibited standard smile-to-smile responding, but only toward displayers low in social power. Such simple mirroring disappeared when high-power perceivers observed high-power displayers of anger. In that case, the opposite response appeared, and perceivers smiled. This pattern of “counter-matching” (smile-to-anger) appeared rather quickly after stimulus onset (between 1 s and 2 s), suggesting that it reflects a spontaneous response, rather than a strategic display. These findings, and related results mentioned earlier, highlight that facial responding in social contexts cannot be solely mediated by low-level motor and affective processes, but rather reflect appraisal of the meaning of the facial display. In the particular case of social power, we assume that to a high-power perceiver, an angry expression by a high-power displayer is interpreted as a competitor’s loss or frustration. Given that “his loss is my gain”, this leads to a positive evaluation of the meaning behind the expression, and, consequently, a smile. This is consistent with suggestions that strategic contexts (competition or cooperation) offer particularly strong social cues to the meaning of the observed expression and modify perceiver’s affective responses (Tamir et al., 2004; de Melo et al., 2014) and facial reactions (Weyers et al., 2009). Accordingly, we used such context manipulation in the current research, specifically to investigate the effects on reactions to android displays.

### Current Research

The goal of the current research was to examine how social context changes human reactions to an android’s “emotional” expressions. As mentioned, one of the key social cues to context is whether an interaction between two agents involves cooperation vs. competition. As an example, consider a simple scenario, assuming no emotional dishonesty and no manipulation, where you, the reader, are playing a bridge game and watching fellow players’ facial expressions directed at you. Your partner’s smile signals a mutual gain, so you will smile. Yet, your opponent’s smile signals your loss, so you will frown. In the current study, we adapted this simple logic to explore how facial responses to android expressions interact with cues to meaning. One theoretical possibility is that spontaneous mimicry will drive human facial responses. If so, we should find smiling to a smile and frowning to a frown even in a competitive context. However, if studies on responses to human facial expressions in strategic contexts are a guide, the more likely theoretical possibility is that participants will respond to the meaning of the android’s facial expressions.

To examine these possibilities, participants played a repeated game. As explained in detail shortly, the context manipulation consisted of telling participants that they are playing with (cooperating) or against (competing) the android. On half of the trials, the android displayed facial reactions (smiles or frowns) to outcomes from the current trial of the game. On the other half of the trials, the outcome of the game was displayed in writing on the computer terminal. This condition was introduced as a preliminary exploration of the possibility that in some social contexts conveying outcome information via facial expressions may be more impactful than conveying similar information via text. We measured participants’ facial reactions using EMG.

## Experiment

### Participants

After screening for missing data and artifacts, the final sample consisted of 27 University of California, San Diego undergraduates (7 females and 20 males). The research protocol was reviewed and approved by the University of California, San Diego Institutional Review Board. Written informed consent was obtained from all subjects.

### Robot Design

This study used an advanced android manufactured by Hanson Robotics (Wu et al., 2009). Its face, made of skin-like materials, can perform facial actions that closely match (in appearance and dynamics) a variety of human expressions, such as happiness and anger (Figure 1). Certified experts on facial expressions programmed the movement for each of Einstein’s 31 face servos (motors), and created a set of actions that matched basic expressions described in Ekman and Friesen’s FACS manual (Ekman and Friesen, 1978). A short video of Einstein displaying both expressions is here: http://pages.ucsd.edu/~pwinkielman/einstein_happy_angry.mov.

### Procedure

Participants were run individually in a large room divided by a partition. On one side of the room was the participants’ desk. On this desk, we located an android and a 17-inch computer screen that displayed task instructions and trial information. The other side of the room contained EMG recording equipment, computers controlling stimulus presentation, Android’s movements and EMG recoding. The position of the android and the computer screen is shown in Figure 1. The participant was seated about 30 inches away from the screen and the android and was able to easily observe either or both, as necessary.

After being introduced to the android, participants responded to a few questions on a computer screen. The key question was to “use a few sentences to describe the android in front of you.” We report these results later as initial impressions of the android. Participants also answered some questions regarding their attitudes about technology, which we will not discuss further (data available upon request).

**Figure 2:**
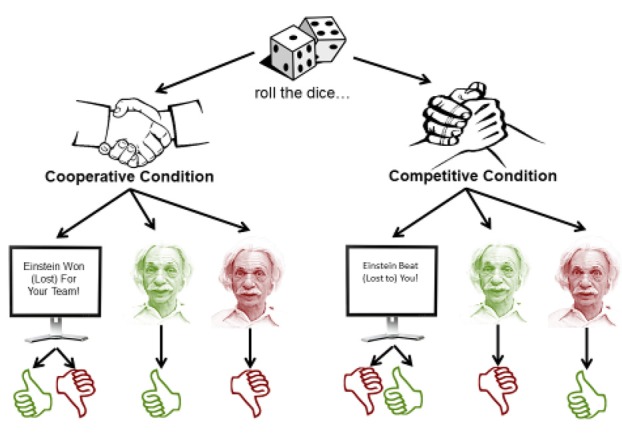
Game task outline. In each trial, a dice game is played. The outcome is conveyed either through the computer screen, or through the android’s expressions. However, what those expressions mean depend on the condition being played. In the cooperative condition, Einstein’s expressions are congruent to the participants’ outcome. In the competitive condition, they are incongruent with the participants’ outcome.

Next, participants proceeded to the game phase of the study. After the instructions, participants played two practice games to ensure that they understood the procedure. The experiment itself consisted of 32 repeated dice games, which depended purely on chance. The overall structure of the game is conveyed in Figure 2. Participants were told that there are two players in the game (the participant and Einstein) and that each player would roll two dice. The sequence of the trial was as follows. The participant always rolled first. This was done virtually, by pressing a button. A dynamic image of two rolling dice (with different numbers of dots flashing randomly) appeared for 1 s, and was then replaced with a 3 s static image showing their resting positions (e.g., a face with 5 dots and a face with 3 dots), see Figure 1. The next screen, shown for 2 s, said that the Einstein is now rolling the dice and again showed a dynamic image of rolling dice, without, however, showing the resting position, or the total outcome of the roll. The dynamic image was then replaced with instructions, shown for 4 s either: (i) look at the screen; or (ii) look at the Einstein’s face. In the screen condition, the outcome information was shown for 6 s by a very simple text (e.g., “Einstein won for your team!”). In the face condition, the outcome information was communicated via the android face. In that case, we gave participants 8 s to orient themselves towards the android and watch its face change (more on that next).

Critically, the game was played under two conditions—one in which Einstein was a competitor, and another in which he was a teammate (see Figure 2). In both conditions, the game outcome depended on whether Einstein’s roll was greater than the participant’s roll. However, if Einstein were a teammate, his win would indicate a win for both Einstein and the participant. If Einstein were a competitor, this would indicate a win for Einstein, and a loss for the participant. This setup ensured that participants would win roughly half of the games they played in either condition. Note again that the numeric outcome of Einstein’s roll was never displayed, just the final trial outcome, making it essential to pay attention to the display of information about the trial outcome.

As just mentioned, participants learned the final outcome of the trial in two ways. On some trials, the information was displayed on the screen in front of them (e.g., “Einstein won for your team!”). On other trials, it was conveyed through Einstein’s facial actions. That is, Einstein would smile if he won and frown if he lost. Participants were told that Einstein’s facial displays are “honest”, in that they are truthful and informative of the outcome of the game. However, notice that depending on the condition, the expressions conveyed different outcomes for the participant. When Einstein plays as a teammate, its smile conveys the participants’ win, and its frown conveys the participant’s loss. However, in the competitive condition, the opposite is true—if Einstein smiles, it means that Einstein won and the participant lost, whereas if Einstein frowns, it means that Einstein lost and the participant won. These different conditions allowed us to test our key questions. First, do people’s emotional facial reactions to the game outcome depend on specific facial actions (smile or frown), or just on the information they convey? Second, do people’s reactions to the game outcome depend on whether the information is conveyed through a facial expression or through a computer screen? After the game phase of the experiment ended, participants were asked several follow-up questions regarding their experience and attitudes towards the robot. Finally, they were debriefed, thanked and dismissed.

### EMG Data Acquisition

EMG was measured by pairs of 4-mm electrodes over the regions of zygomaticus major (cheek) and corrugator supercilii (brow), in accordance with the EMG processing standards (Tassinary and Cacioppo, 2000). For the zygomaticus major muscle, the first electrode was placed in the middle of an imaginary line between the lip corner at rest, and the point where the jaws meet (approximately near the ear lobe). The second electrode was placed a collar width (approximately 1 cm) posterior to the first. For the corrugator supercilli muscle, the first electrode was placed right above the eyebrow, on an invisible vertical line from the corner of the eye up. The second electrode was placed a collar width posterior to the first (following the eyebrow arch).

AcqKnowledge software (Biopac Systems, Goleta, CA, USA) along with Biopac (Biopac Systems, Goleta, CA, USA) were employed to acquire the EMG signal. Figure 1 shows the approximate location of the electrodes and how they correspond to the Android’s face. The amplified EMG signals were filtered online with a low-pass of 500 Hz and a high-pass of 10 Hz, sampled at a rate of 2000 Hz, and then integrated and rectified using Mindware EMG software, version 2.52 (MindWare Technologies Ltd., Columbus, OH, USA). EMG data was recorded for 6 s following the onset of display of the game’s outcome on the screen, or the onset of Einstein’s expression.

### EMG Data Processing

EMG data was analyzed using MATLAB (version R2011b, The Mathworks, Natick, MA, USA), JMP (version 10, SAS Institute Inc., Cary, NC, USA), and SPSS (version 20, IBM Corporation, Armonk, NY, USA). EMG data from the practice block and the recognition block was excluded from the rest of the analyses, due to the amount of noise they introduced when standardizing activity. Data was first averaged in 500 ms intervals across a trial (i.e., 12 data points for a 6 s trial). Extreme values (values greater than 3 standard deviations away from the mean) were excluded from the analysis. Next, data was standardized within participant and within each muscle. A median of the activity during a time window of 2000 ms before the outcome was displayed served as a baseline. We calculated baseline-corrected activity for each participant and each muscle across the 6 s time period that began when information was either displayed on the screen, or a command was sent to Einstein to express the outcome (either smiling, or frowning). Activity was averaged in 500 ms chunks, totaling 12 timepoints across the 6 s trial.

## Results

### Initial Impressions of the Android

**Figure 3:**
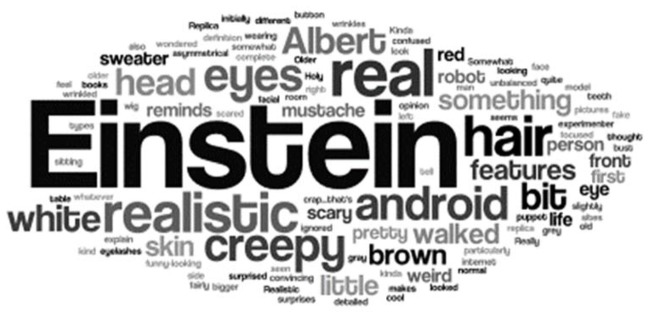
Word cloud of spontaneous descriptions of our android. Participants were asked to freely describe the “android in front of you”. Most participants mentioned similarity to Einstein and visual features (mustache, wrinkles, etc.). Interestingly, 61% mentioned how realistic seemed, and a surprising 34% mentioned negative emotional terms such as “creepy” or “scary”.

As mentioned in the procedure section, before starting the game, participants were asked to describe in their own words “the android in front of you” (see Figure 3 for a word cloud of the terms used in these descriptions). Frequency analysis revealed that participants find Einstein highly realistic: 61% used exact words or close synonyms of “realistic”, “real”, “human-like”, “life-like” and “person”. Spontaneous descriptions of 34% of the participants imply unease about Einstein and include words, or close synonyms of “scary”, “creepy”, or “weird”.

### Predictions and Analysis Strategy

The analyses explored the following predictions. First, we examined participants’ facial reactions to the trial outcome (win vs. loss). We expected stronger zygomaticus (smiling) activity after winning than losing, and stronger corrugator (frowning) activity after losing than winning. Second, we examined whether the effects of the trial outcome depended on how it is conveyed. We expected that participants would respond according to the value of the trial outcome (win vs. loss), regardless of whether it is communicated with congruent or incongruent android expressions (e.g., win communicated with a smile or a frown). Third, we predicted stronger EMG reactions to the value of the outcome when it was communicated by Einstein’s expressions, rather than the computer.

In order to examine these questions, we used mixed effects models on the zygomaticus and corrugator EMG activity with Subject as a random effect, and other factors (listed below) as fixed effects. We used two main statistical models of EMG activity. The first model takes data from the entire trial—all 6 s, broken down by half-second chunks (so 12 time points of 500 ms each). Since this model represents the entire trial period, it captures how EMG activity changes over time. However, it is also important to note that it takes the android about 1 s to initiate the expression, and that its smile lasts about 2 s and its frown lasts about 3 s, please see Figures 5, 6 in our previous publication on this android (Hofree et al., 2014). In addition, in a previous study with this android, we observed that the lag between android and participant expressions was on average 1.18 s. Thus, unlike many documented facial reactions to briefly presented static and fully developed expression pictures, which appear rapidly (1–2 s), the full impact of the android’s dynamic expression should appear primarily in the later portion of the trial.

It is also well known in facial EMG literature that emotion-driven (rather than mimicry-driven) differences occur after a delay of 1 or 2 s from the stimulus onset. Thus, we also used a second statistical model that takes into consideration the average EMG activity during the last 3 s (second half) of the trial, accounting for the time it takes for Einstein, and then the participant, to produce expressions. In sum, the first statistical model, which we will refer as the “Time Averages Model,” allows more fine-grained analysis of how the reactions developed across time, whereas the second statistical model, which we will call the “Late Averages Model,” simplifies the analyses of main effects in the key time period. Finally, because there is still a considerable debate on how to properly calculate effect sizes in repeated-measure analyses, we provide variability estimates in the result figures (Lakens, 2013).

### Participants’ Expressions Reflect the Meaning of Einstein’s Facial Actions

As mentioned, our key question was how participants’ expressions depended on game outcome and social context when Einstein conveyed this information via facial actions. To do so, in this section the analyses focus on data only from trials where the android (but not the computer) conveyed the results of the game. We first discuss the overall effects of the game outcome, regardless of whether the outcome occurred in the cooperative block or the competitive block. We then discuss the effects of context—cooperative vs. competitive block. We return to the difference between expression-based and text-based outcome information later in the section “Reactions to Game Outcome When it is Conveyed by the Android or the Computer.”

#### Effects of Game Outcome

**Figure 4:**
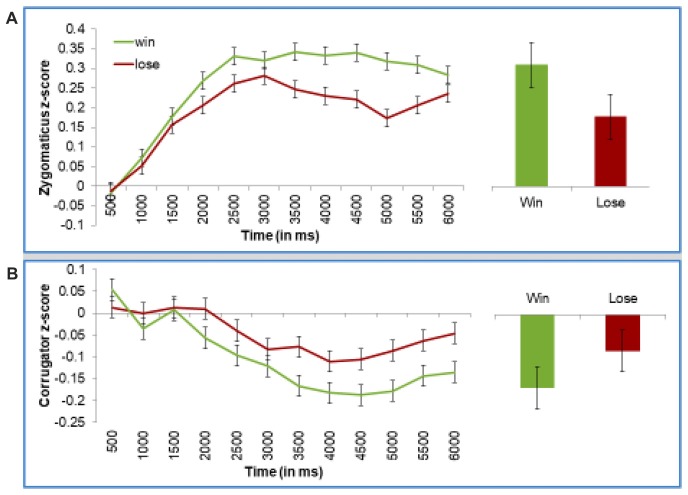
Participants smile more when winning, and frown more when losing, when the android conveys the outcome of the game. Panel **(A)** presents z-scored EMG activity in the zygomaticus muscle and panel **(B)** presents z-scored EMG activity in the corrugator muscle. Both present activity across the length of the trial, or during the 2nd half of the trial, where emotion-driven differences were expected to occur. Error bars represent the standard error of the subject means.

As can be seen in Figure 4, participants smiled more when they won (Figure 4A). The corrugator muscle slowly relaxed over the trial, but participants showed less relaxation when they lost (Figure 4B). This “relaxation” pattern probably reflects that valence effects sometimes result in difference in corrugator relaxation, especially since at baseline, corrugator activity could be enhanced in anticipation or concentration before the trial outcome.

For statistical tests, we first discuss the results of our “Time Averages Model” model, which looks at mean values for each of the 12 time points. Specifically, we conducted a mixed model regression analysis on EMG activity with Outcome (win vs. loss) and Time (12) as fixed effects, and Subject as a random effect. For simplicity, this model was run separately on the zygomaticus and corrugator muscle.

On the zygomaticus (Figure 4A), we found an interaction between Time and Outcome, reflecting that in the later portion of the trial, zygomaticus activity increases more to wining than losing, *F*_(1,10755)_ = 8.47, *p* = 0.004. On the corrugator (Figure 4B), we also found an interaction of Time and Outcome, reflecting that activity in the later portion of the trial decreases less to losing than winning, *F*_(1,10792)_ = 5.27, *p* = 0.02.

Using our “Late Averages Model” we confirmed that these effects were robust in the second half of the trial. This was statistically tested using a mixed model regression on mean EMG activity in the second half of the trial, with Trial Outcome as a fixed effect and Subject as a random effect. Zygomaticus activity was significantly stronger when the participant won than lost, *F*_(1,894.7)_ = 5.088, *p* = 0.02. Corrugator activity showed a trend in the same direction, *F*_(1,888.2)_ = 2.46, *p* = 0.12, which is consistent with the statistically robust findings from the “Time Averages Model”.

#### Effects of Social Context: Competitive vs. Cooperative Interaction

**Figure 5:**
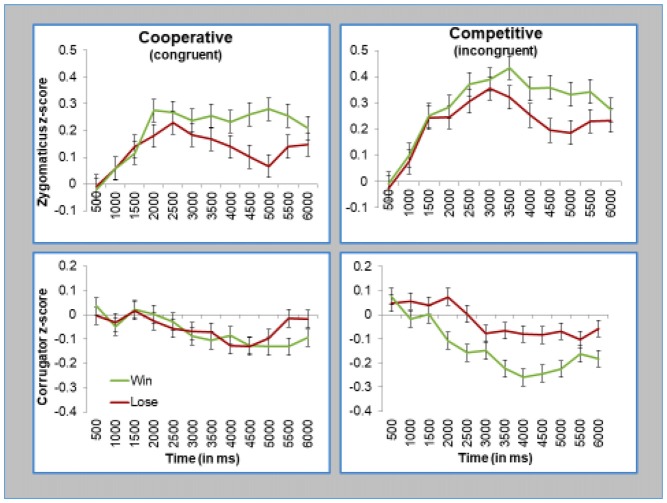
Participants smiled when they won, regardless whether Einstein smiled (congruent) or frowned (incongruent). Participants frowned when they lost, regardless whether Einstein frowned (congruent) or smiled (smiled). Top panels presents z-scored EMG activity in the zygomaticus muscle, and bottom panels presents z-scored EMG activity in the corrugator muscle. Left panels present data during the cooperative condition, and the right panels present data during the competitive condition. Error bars represent standard errors of the subject means.

As mentioned earlier, our key question was how the effects of the game outcome depended on whether it is conveyed through a smile or a frown. This question can be answered by testing whether or not participants responded to the game outcome differently in the cooperative block (where smiles signal positive game outcomes and frowns signal negative outcomes, thus, making expression and outcome “congruent”) and competitive block (where smiles signal negative outcomes and frowns signal positive outcomes, thus, making expression and outcome “incongruent”). As shown in Figure 5, participants facial reactions reflected the valence of their outcome. This is true even when the outcome was conveyed via Einstein’s incongruent facial actions, as was the case during the competitive block (as shown in the right panels of Figure 5).

Once again, we formally tested the effects with our two statistical models. First, we ran the initial “Time Averages Model”, which was a mixed model regression analysis on EMG activity with Block (competitive vs. cooperative), Trial Outcome, and Time as fixed effects and Subject as a random effect. Again, each muscle was tested separately. This analysis replicated the key effects of the Outcome, as described above. Critically, we found no significant interaction on Block × Outcome (zygomaticus, *F*_(1,71.6)_ = 0.55, *p* = 0.46, corrugator, *F*_(1,49.5)_ = 1.42, *p* = 0.24), or Block × Outcome × Time (zygomaticus, *F*_(1,20164)_ = 1.8, *p* = 0.18, corrugator, *F*_(1,20180)_ = 1.74, *p* = 0.19). In short, this analysis showed that the block type (competitive vs. cooperative) did not moderate the reactions to outcome or influence the time it took for these reactions to surface.

Our second statistical model, focusing on mean activity during the second half of trial, confirmed these results. Once again, we found no evidence that the block type (competitive vs. cooperative) influenced participants’ reactions to outcome (test for interaction of Outcome × Block on zygomaticus, *F* = 0.18, n.s.; corrugator, *F* = 1.7, n.s.). Overall, these analyses demonstrate that participants smile more to wins and frown more to losses, regardless of whether the android conveys this information through congruent or incongruent expressions.

#### Reactions to Game Outcome When It Is Conveyed by the Android or the Computer

As we just described, participants showed robust reactions to game outcomes conveyed through Einstein’s expressions. This answers the main question of the study. It is also interesting to test if conveying outcomes via the face differs in its effects from conveying that same information via text on a computer (we address the limitation of our “text” control condition in the general discussion). To test for these subtler effects, we used our “Time Averages” model and conducted an analysis with Information Display Mode (android vs. computer), Trial Outcome (win vs. lose), and Time as fixed effects, with Subject as a random effect. All the EMG analyses were done separately for each muscle.

**Figure 6:**
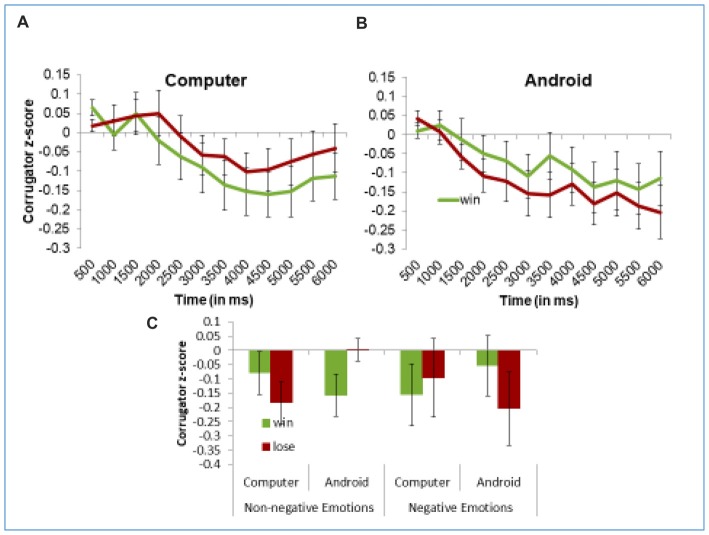
Participants frown to losses more than wins when the information is conveyed through the android’s expressions **(A)**, and not through the computer screen **(B)**. This sensitivity to the way the outcome is presented is dampened when participants express negative emotions in their initial impression of Einstein **(C)**. Panels **(A,B)** present z-scored EMG activity in the corrugator across the trial. Panel **(C)** demonstrate mean z-scored EMG activity in the corrugator muscle in the 2nd half of the trial. Error bars represent the standard error the subject means.

On the zygomaticus, our “Time Averages” analysis replicated the key findings described earlier in the section on outcome effects, but found no effects of Information Mode. This means that participants’ smiling did not differ when Einstein’s face conveyed information than when it was written on the computer screen. However, on the corrugator, our “Time Averages Model” found a significant Information Mode × Trial Outcome × Time interaction, *F*_(1,20192)_ = 4.47, *p* = 0.03. Figure 6 shows these results. To better understand this interaction, we ran separate models for each type of Information Mode. As shown in the left panel of Figure 6, when trial results are conveyed through the computer, the Outcome does not influence corrugator EMG activity, as reflected by the non-significant Outcome × Time interaction, *F*_(1,9468.3)_ = 0.77, *p* = 0.38. However, when Einstein expresses the outcome (right panel of Figure 6), we find a significant Trial Outcome × Time interaction, *F*_(1,10792)_ = 5.27, *p* = 0.02 (same result as described earlier for analyses by outcome). Interestingly, our “Late Averages Model” found no significant effects involving Information Mode, perhaps because this analysis is restricted to only later portion of the trial. Still, these results offer some preliminary evidence that participants react stronger to the trial outcome, at least on the corrugator muscle, when this information is conveyed by Einstein’s facial action than by computer screen.

We also wanted to explore whether differences in the way the information is conveyed influenced reactions to competitive and cooperative games. For this reason, we ran our “Time Averages Model” and ran regression analyses with Block (cooperative vs. competitive) × Information Display Mode (android vs. computer) × Trial Outcome (win vs. loss) × Time as fixed effects, and subjects as random effects. Interestingly, in addition to replicating the effects of Trial Outcome as mentioned above, we found a significant Block × Information Mode × Time interaction (*F*_(11,289.4)_ = 2.32, *p* < 0.01) in the zygomaticus muscle (no effects of Block were found in the corrugator). In order to further interpret this interaction, we ran separate analyses for the zygomaticus for the trials in which Einstein conveyed the trial outcome, and those in which the computer conveyed the same information. We found that when Einstein expressed the outcome, the zygomaticus was more active overall during trials in which Einstein was a competitor, than when he was on the same team, as is demonstrated by a main effect of Block in these trials (*M*_competitive_ = 0.27, *M*_cooperative_ = 0.17, *F*_(1,25.8)_ = 4.75, *p* = 0.04). However, no such effects were found when the computer conveyed the information. Thus, it appears that learning about the outcome of a trial through Einstein’s expressions elicits more zygomaticus activity in competitive situations. This could be due to a more positive reaction to competition vs. cooperation, which is intensified when expressed through a present opponent.

## Discussion

The current study examined people’s reactions to facial actions of an advanced android, and how these reactions depend on the context in which these facial actions appear. The key results of this study show that in a game context, human responses to the android’s facial actions are primarily driven by their informational value. That is, a “smile” by a cooperative android and a “frown” by a competitive android trigger a comparable amount of human smiling. Similarly, a “frown” by a cooperative android and a “smile” by a competitive android elicit a comparable amount of human frowning. These findings make sense given that in the current study, the context (an interactive game) clearly specified the nature of the information communicated by android’s facial display. The cooperative context made clear that a “smile” (or frown) is good (or bad) news, and the competitive context made clear that smile (or frown) is bad (or good) news. As such, these findings are theoretically compatible with accounts of facial expressions that emphasize their informative functions, greater impact than similar content conveyed and highlight the power of contextual modulations of expression meaning (Russell et al., 2003; Niedenthal et al., 2010; Hess and Fischer, 2013; Seibt et al., 2015).

Importantly, these findings go beyond earlier demonstrations of responses to facial displays of the same android under spontaneous observation—a relatively context-free situation. As mentioned in the introduction, when participants are asked to simply watch the android in the same room, they spontaneously match its facial displays, smiling to “smiles” and frowning to “frowns” (Hofree et al., 2014). This suggests that in a default context, realistic androids can drive human responses via basic mirroring mechanisms, and in richer social contexts, they can drive human responses via more appraisal-like mechanisms. One suggestion for future studies would be to directly compare the speed and effort involved in production of facial expressions to the android in both contexts–mere observation vs. observation in a richer social context. This would be interesting, especially given the recent studies on reactions to human facial expressions suggesting that social context can be incorporated even in fast, spontaneous reactions (Carr et al., 2014). Methods, like EEG, that offer more precise timing of relevant brain processes might be particularly helpful here (Jin et al., 2014).

Another idea for a future study is to directly compare the responses to an android and another human placed in cooperative and competitive settings. Some of our earlier work on context-free, mere observation of facial actions, as well as gestures made be an android and a human, suggests that the results are quite similar (Hofree et al., 2014, 2015). However, in a strategic context, human participants may find it easier to emotionally detach from an android than another human being and only focus on the meaning of facial action displayed by the android. Anecdotally, it seems hard to resist a salesperson’s smile even when we are fully aware of the competitive nature of the relationship. This anecdotal observation fits with research studies showing the impact of automatic imitation processes even in a strategic context (Cook et al., 2012). But again, other research with human expressions in a strategic context suggests that people can respond primarily to their meaning, when required by the situation (Tamir et al., 2004; Weyers et al., 2009; de Melo et al., 2014).

Another interesting, but preliminary finding from the current study suggests that the same outcome signaled by the android’s facial action, rather than communicated via computer screen, elicited greater facial reactions, at least on the corrugator muscle. It is worth nothing here that the corrugator often provides a more sensitive index of both mimicry and affective reactions (Hess et al., 2017), albeit many contextual modification effects have been observed on the zygomaticus, which is under more voluntary control (Niedenthal et al., 2010). Setting aside these complications, the preliminary finding of a difference between expressions and text is consistent with evidence that information conveyed via body-compatible means is easier to process and has greater impact (Siakaluk et al., 2008) as well as with evidence that evaluative content presented via pictures, has greater impact than similar content conveyed by words (Winkielman and Gogolushko, 2018). Specifically, communicating the same information via facial actions can draw on the same mechanisms that are involved in the production of facial action under emotional state (for reviews, see Niedenthal et al., 2010; Winkielman et al., 2015). Of course, faces are also more interesting stimuli than computer screens and can spontaneously draw and hold attention, so future studies may want to assess the role of such factors. It is also necessary to acknowledge the limitation of our design in terms of making this comparison. For one, our android’s facial expression not only conveys the evaluative outcome but also an “emotional” reaction to the outcome, whereas text only conveys the evaluative outcome (win/loss). Another limitation of our design is that the face display condition is more socially engaging than the computer text condition. This is because the same agent (Android) that wins or loses states the outcome, whereas in the computer text condition, the outcome is announced by a different, less involved agent. Future studies could address this issue by having the android announce the outcome of the trial only verbally (without any facial action). All these differences may explain why other studies that compared facial displays with textual information, which also had information about reactions, found no differences between faces and texts (de Melo et al., 2014). More generally, it is worth noting that studies involving facial EMG, like ours, are less suited than EEG studies for detecting the amplitude, timing and brain processes of subtle reactions to facial and textual displays (Jin et al., 2012, 2014).

It is also worth mentioning that the current findings were obtained even though 34% of the participants felt uneasy about the android, characterizing him as “scary”, “creepy”, or “weird”. In fact, preliminary analyses suggested that these participants with negative impressions were relatively less influenced by the android’s facial actions, see Figure 6C, analyses on request. At the same time, most (61%) of participants found the android highly realistic. These findings suggest that high realism, rather than positive affect, determine the ability of the android to successfully communicate emotional states. This possibility has some intriguing implication for the uncanny valley hypothesis (Mori, 1970; Cheetham et al., 2011; Carr et al., 2017). It suggests that a highly realistic agent with anthropomorphic appearance may have a negative impact on human’s overall evaluative response, but at the same time may be able to resonate with and impact humans via its “expressive” movements (Hofree et al., 2014) as well as actions and gestures (Hofree et al., 2015).

Finally, it is worth discussing some implications of the study regarding the impact of advancing technologies on human experience and behavior. It is clear that agents featuring high realism in anthropomorphic design are entering daily life in fields like education, entertainment, healthcare, marketing and the hospitality industry. These advanced agents look progressively more human-like and they act progressively more human-like, including their emotional displays (Gratch et al., 2013). The current study suggests the success of these technologies in effective emotional signaling, but raises new challenges to understand how such agents’ emotional displays interact with social context in determining human emotional experience and behavior.

## Author Contributions

GH, PR, MSB and PW developed the study idea. GH and AR ran the study. GH performed the analyses. GH and PW wrote the manuscript.

## Conflict of Interest Statement

The authors declare that the research was conducted in the absence of any commercial or financial relationships that could be construed as a potential conflict of interest.
